# Use of GWAS analysis in deciphering the inability of barley seeds to germinate after hypoxia

**DOI:** 10.1093/jxb/erad198

**Published:** 2023-08-03

**Authors:** Kurt V Fagerstedt

**Affiliations:** University of Helsinki, Faculty of Biological and Environmental Sciences, Organismal and Evolutionary Biology Research Programme, FI-00014 University of Helsinki, Finland

**Keywords:** Barley, genome-wide association analysis, germination, hypoxia, laccase, lignification, secondary dormancy, submergence

## Abstract

This article comments on:

**Gómez-Álvarez EM, Tondelli A, Nghi KN, Voloboeva V, Giordano G, Valè G, Perata P, Pucciariello C.** 2023. The inability of barley to germinate after submergence depends on hypoxia-induced secondary dormancy. Journal of Experimental Botany **74**, 4277–4289

**The important crop species barley, *Hordeum vulgare*, is prone to flooding stress and has difficulties germinating under hypoxic conditions. Gómez-Álvarez *et al.* (2023) investigated a large collection of barley cultivars and their ability to germinate after short-term submergence stress followed by recovery. In a simple experimental setup that tested precisely the germination ability of barley grains, and a complex post-analysis, they have concluded, among many other matters, that inability to germinate is largely due to a state of secondary dormancy activated under submergence. The dormancy is probably driven by a more lignified seed coat, a trait that takes place during grain development. The genome-wide association analysis (GWAS) was the key to find out the trait responsible for the submergence stress tolerance and recovery in barley grain.**

A recent review article by Fukao *et al*. (2019) points out the importance of understanding plant responses to submergence stress, and states that on a global scale, losses of billions of dollars have been caused by floods affecting crops (Food and Agriculture Organization of the United Nations, 2017). The second report in 2021 places further emphasis on this fact and shows data on increasing flooding events due to the temperature rise caused by climate change (Food and Agriculture Organization of the United Nations, 2021). Research on flooding tolerance of crop plants has been conducted for decades, and attempts to unravel the role of the physiological, anatomical, morphological, and genetic basis of flooding tolerance have largely concentrated on individual pre-selected traits that seemingly may have had an important role in overcoming hypoxic stress. In most cases, these studies could not show whether some other feature could have affected the flooding tolerance of the specimens at the same time.

Gómez-Álvarez *et al*. (2023) have taken a different approach. First, they selected representative barley lines from the vast WHEALBI barley collection and carefully created stress conditions for germination under submergence and recovery with and without feeding of sucrose, glucose, and mannitol (Fig. 1). Additional experiments with abscisic acid (ABA) quantification and supplementation with nitric oxide (NO) donors and scavengers were done on a subgroup of representative accessions. The results showed that in submergence-sensitive seeds there is lower capacity for oxygen diffusion, higher alcohol dehydrogenase 1 (ADH1) activity, higher expression of ABA-insensitive 5 (ABI5), and sensitivity to NO feeding, which all point to induced dormancy. Most importantly, the authors combined this study with a GWAS of the barley accessions (tolerant or intolerant to hypoxic germination) and identified the most significant single nucleotide polymorphisms (SNPs) associated with the trait to be located within a laccase gene (*LAC*; Fig. 1).The *LAC* coding sequences in the tolerant and intolerant barley lines did not show any differences but, very interestingly, their promoter sequences were partially or totally deleted in the tolerant barley accessions. Further analyses of the whole plant at different developmental stages revealed that in the sensitive accessions, *LAC* expression was high at the last maturation phase of the developing kernel, causing higher lignification, and hence slower diffusion of O_2_ into the germinating grain. In brief, in the sensitive accessions, the inability to germinate and induced dormancy are due to a more lignified seed coat and hence reduced oxygen availability. This study shows convincingly that combining traditional stress sensitivity assays and gene expression studies with GWAS can be a powerful approach. The excellent results are enabled by the fact that the exome SNP coverage is dense in the many barley accessions that are available at the WHEALBI collection (Fig. 2).

**Fig.1.**
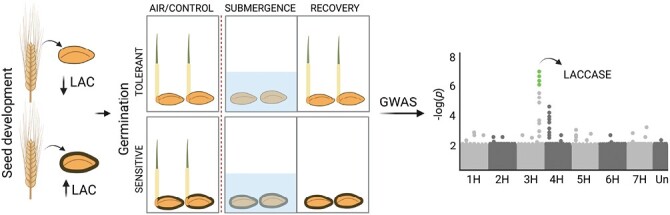
Gomez Alvarez *et al*. (2023) tested grains of *Hordeum vulgare* ssp. *vulgare* accessions, belonging to the WHEALBI collection, for germination under air/control conditions, and under submergence (2 d) and then recovery for 5 d. GWAS analysis showed that expression of a chromosome 3H *laccase* gene correlated positively with the sensitivity to submergence. Careful subsequent studies showed that the tolerant accession had a lower lignification level of the seed coat, leading to impaired diffusion of oxygen into the grain and, hence, lower recovery from the submergence stress (figure created with BioRender.com).

**Fig. 2.**
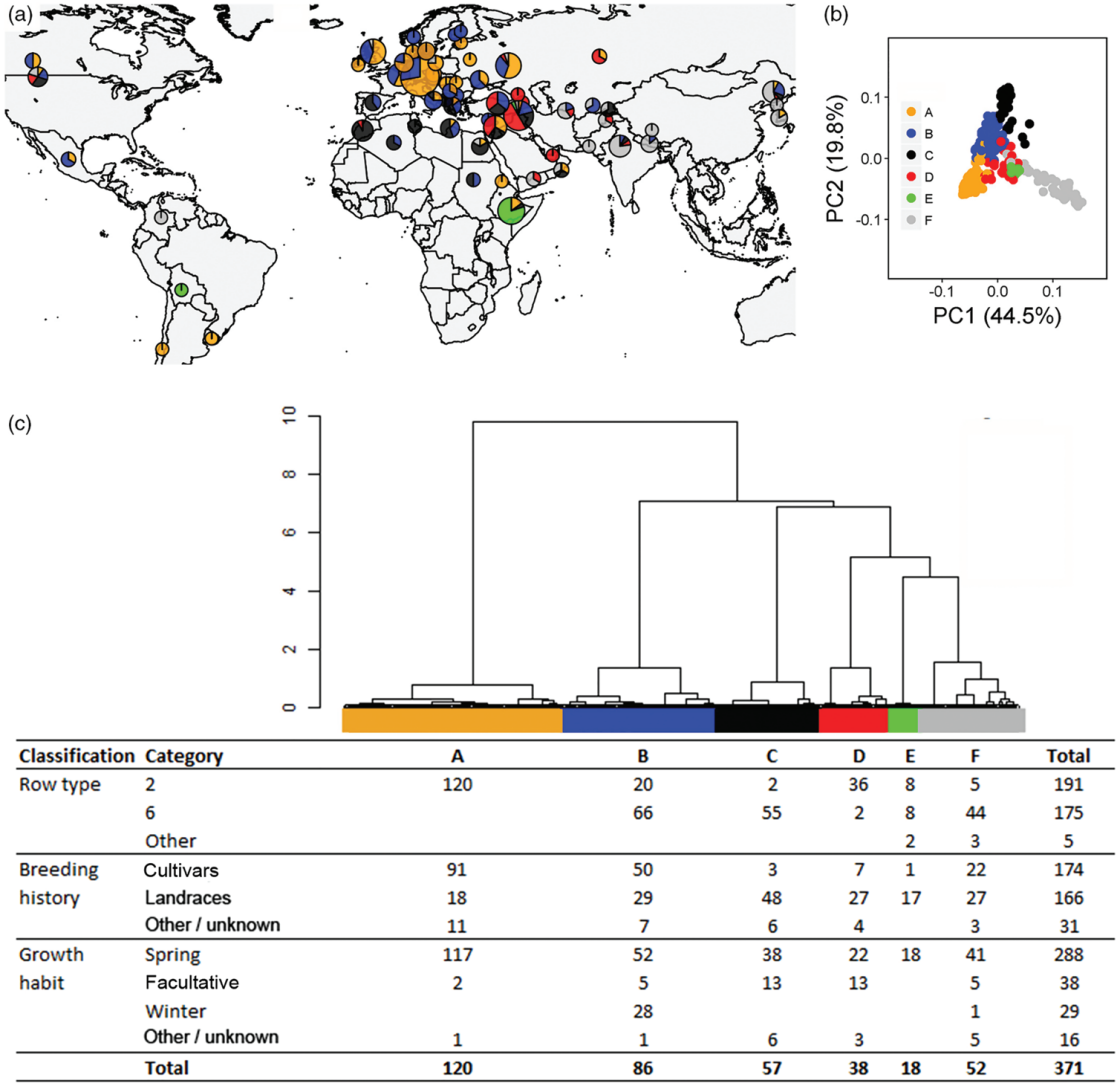
The WHEALBI collection (https://www.whealbi.eu/). Within the European Union 7th Framework Programme for Research (FP7), the WHEALBI project was launched in January 2014. It is coordinated by the Institut National de la Recherche Agronomique (INRA, France) and involves the best European genomic or agronomy laboratories working on wheat and barley. The objective of the project is to strengthen bread wheat and barley production in Europe by creating a collection of wheat and barley cultivars and landraces, and a database with exome deep sequencing and dense SNPs, to enable the development of new productive wheat and barley varieties. The figures show the barley collection of 371 accessions from all over the world (A) with distinct genetic variation in six barley subpopulations (B and C). The colours in the pie-charts correspond to the principal component analysis (B), and the slice size shows the proportion of each genotype in the different geographical areas. The hierarchical clustering (Ward distances) indicates the division of six subpopulations according to row type, breeding history, and growth habit (data with permission from Bustos-Korts *et al*., 2019).

## Benefits of GWAS analysis and future perspectives

Within the last few years, we have witnessed an increasing number of studies where GWAS has been used in research on barley, especially related to abiotic stress tolerance (Bustos-Korts *et al*., 2019; Alqudah *et al.*, 2020; Karunarathne *et al*., 2020; Tsai *et al*., 2020; Abou-Elfawa and Shehzad, 2021; Borrego-Benjumea *et al*., 2021). Combined with field trials and/or greenhouse tests, GWAS is indeed a powerful technique to get to grips with the genetic basis of stress tolerance. In particular, if we have a database of dense SNPs, as is the case in the work of Gómez-Álvarez *et al*. (2023) using the WHEALBI database, we can get information on the expression of a relatively small number of candidate genes that most probably are responsible for the trait we are looking for.

Research on flooding and submergence tolerance in plants has advanced rapidly in the last 10 years. We now have information available on individual metabolic pathways and their regulation (Xiong *et al.*, 2021), on the regulation of core hypoxia-responsive gene expression (Mustroph *et al*., 2010; Licausi *et al*., 2011b; Sasidharan and Mustroph, 2011), on the perception of oxygen concentration in the tissues (Gibbs *et al*., 2011; Licausi *et al*., 2011a) and its effects on the roles of NO and phytoglobins in metabolic regulation (Hartman *et al*., 2019; Gupta *et al*., 2021), on signalling crosstalk within a plant on the transfer from dry to wet habitats, as well as on the recovery phase, as reviewed in Fukao *et al*. (2019). We are still lacking information on the regulation of flooding tolerance (or lack thereof) in many individual crop species, but this information is accumulating quickly. In addition, we have already witnessed examples where all this knowledge at the molecular level is put into practical use. An excellent example ofthis work is the development of new flooding-tolerant, and incidentally also drought-tolerant, rice cultivars in which the *sub1A* allele has been introduced (Fukao *et al*., 2011). As climate change is advancing and bringing increased events of both flooding and drought, it would be wise to place more effort on research on multiple stress tolerance in future crop cultivars.

## The WHEALBI collection

The WHEALBI collection of the WHEALBI project is a European Union 7th framework project started in 2014 and is led by INRA in France by the WHEALBI scientific coordinator Gilles Charmet and WHEALBI communication leader Sébastien Crépieux of ARCADIA International in Belgium. The full name of the collection is Wheat and Barley Legacy for Breeding Improvement. The project includes 18 partner institutions in Europe and one in Israel. There are 511 barley accessions in the collection that have come from countries covering the whole world (Fig. 2). The aim of the project is to provide >1000 wheat and barley accessions for researchers and breeders. The wheat and barley lines have been collected globally with a focus on germplasm more adapted to European conditions, and the barley collections contains two- and six-row cultivars, landraces, and accessions of spring and winter growth habits. The database includes exome deep sequences of all the accessions, with 450 000 SNPs having been identified in barley genomes. In addition, phenotypic data for a subset of the accessions are available as well as lists of candidate genes and alleles for key traits including grain quality, resistance against fungal diseases, and frost and drought tolerance. The project ended in 2019 but all the data are kept alive and used by an increasing number of researchers and breeders.

## Key findings

This current study demonstrates that GWAS is a powerful tool to identify useful traits as important targets for breeding stress tolerance to crop species. Gómez-Álvarez *et al*. (2023) highlight an approach with careful planning of the stress experiments to pinpoint particular features in the stress response. This was eventually combined with gene expression of a small number of genes enabled by a dense coverage of SNPs in the GWAS analysis. The work draws attention to the *LAC* gene as a new target for genetic manipulation for better seed survival under hypoxia, a result that could also be applied to breeding of other cereals.
